# Supplementary material for: The Two-Component System 09 of Streptococcus pneumoniae Is Important for Metabolic Fitness and Resistance during Dissemination in the Host
Source: Microorganisms. 2021 Jun 23;9(7):1365. doi: 10.3390/microorganisms9071365 (PMC8306541; doi:10.3390/microorganisms9071365)
Supplement: Supplementary file 1 [file microorganisms-09-01365-s001.zip › microorganisms-1253919-supplementary.pdf]

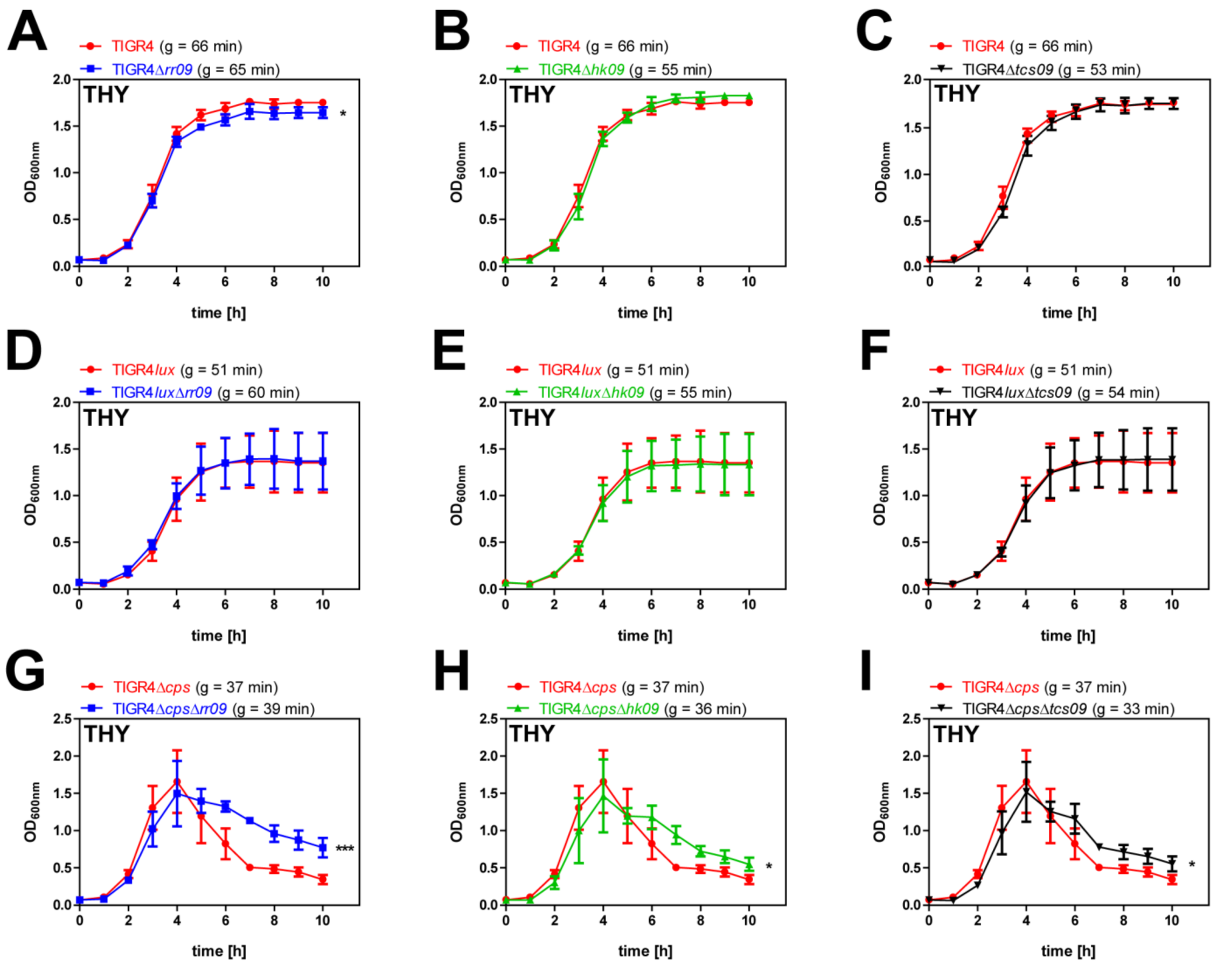

Figure S1: **G**rowth behavior of RR09-, HK09- and TCS09-deficient pneumococci and its parental strains TIGR4, TIGR4*lux* or TIGR4Δ*cps* in THY-medium. Isogenic *tcs09*-mutants and parental strains TIGR4 (**A-C**), TIGR4*lux* (**D-F**) or TIGR4Δ*cps* (**G-I**) were cultivated in THY-medium at 37°C under microaerophilic conditions without agitation. Results are presented as the mean ±SD for three independent experiments. The mean value of the doubling time (g) from three biological replicates of the respective strain is provided. A two-way Anova proved a significance with p-value * < 0.05 and *** < 0.001 relative to the parental pneumococcal strain.

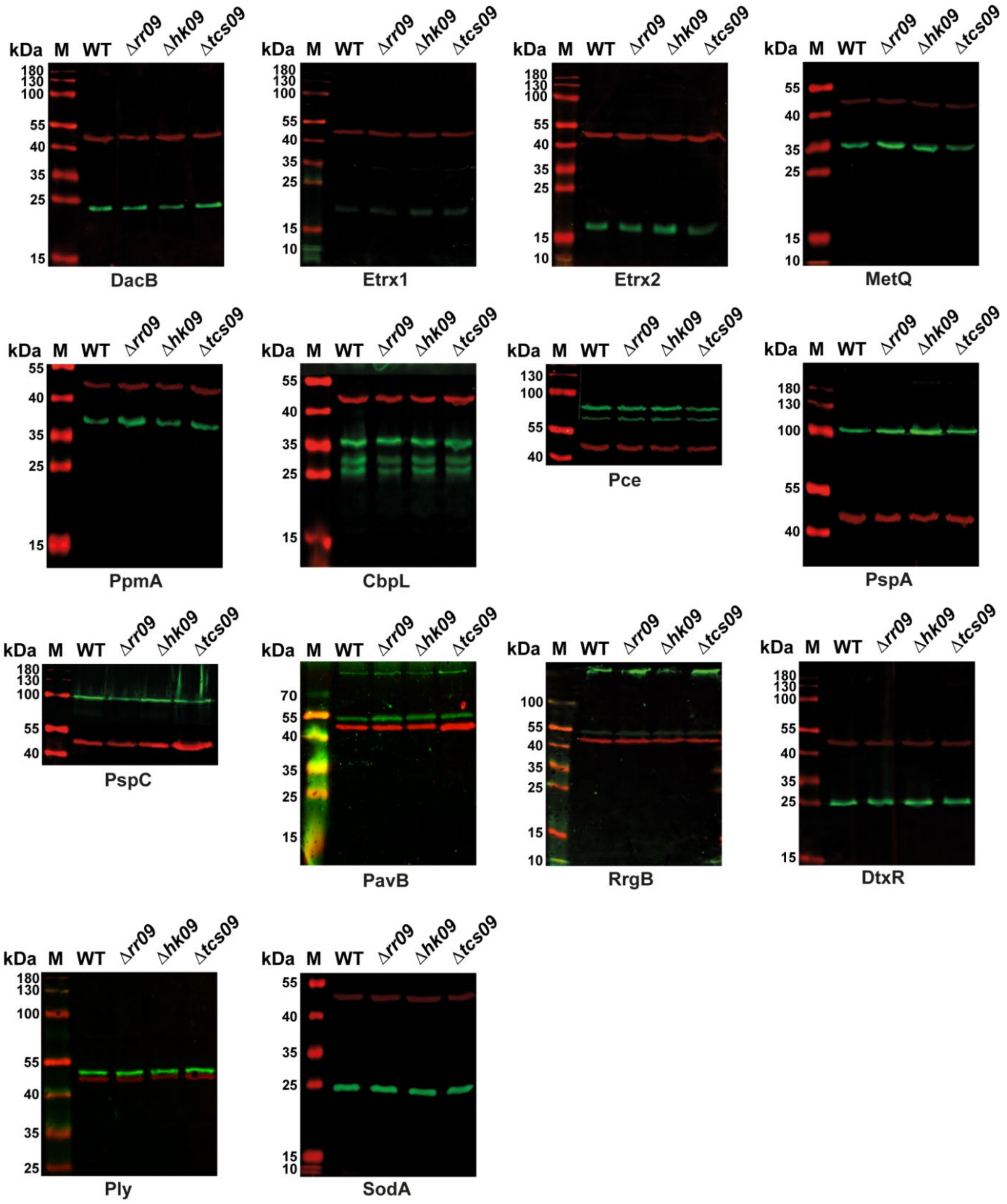

Figure S2: Impact of TCS09 on pneumococcal virulence factor expression. The pneumococcal *rr09- hk09-* and *tcs09*-mutants of TIGR4Δ*cps* were analyzed for lipoprotein, choline-binding protein, sortase-anchored protein and intracellular protein expression, respectively. Protein expression of selected candidates was detected with polyclonal protein-specific antibodies (1:500) generated in mice followed by IRDye® 800CW fluorescence-coupled secondary antibody (1:15000; green). Detection of Enolase (47 kDa) as loading control was performed with rabbit anti-Eno antibody (1:12,500) and IRDye® 680RD fluorescence coupled secondary antibody (1:15000; red). Scanning of the immunoblots was conducted with Odyssey® CLx Scanner.

CI values

The following tables provide a list of all calculated competitive index (CI) values for Figure 8. CI values were calculated using the following formula:

$$CI = \frac{Mutant\ x\ (CFU\ x\ 10^6)}{Wild - type\ x\ (CFU\ x\ 10^6)}$$

Table S1. Calculation of the Competitive Index – nasopharyngeal cavity

**TIGR4*lux* vs TIGR4*lux*Δ*rr09***		**TIGR4*lux* vs TIGR4*lux*Δ*hk09***		**TIGR4*lux* vs TIGR4*lux*Δ*tcs09***	
**24 h**	**48 h**	**24 h**	**48 h**	**24 h**	**48 h**
0.673075	0.901638	4.846191	1.159096	1.459463	1.071431
0.584699	1.140347	0.553030	1.148144	1.264147	0.897107
1.337075	1.119656	1.127265	0.987343	1.000000	0.999994
0.617355	0.662339	1.000000	1.060600	0.746031	0.884615
1.051277	3.40916	1.023807	0.923079	1.027775	0.600719
0.156862	1.219297	1.172406	0.729732	1.184217	0.781816
1.634408	1.046875	1.108698	0.842105	1.096154	0.835165
1.205869	1.563636	0.518987	2.560006	0.617021	1.322566
0.982858	1.303027	1.759995	1.426664	1.000000	0.777778
0.363636	0.464843	1.234567	2.595236	0.610386	0.686567
0.962025	1.283779	0.501155	0.687495	1.714286	0.452784
0.865671	1.395605	1.617016	2.369226	0.530249	1.133333
1.077918	2.882330	0.567568	0.651027	0.753558	0.956898
0.776316	1.655175	0.569890	0.662870	0.617250	0.563981
**Median:**					
**0.9138**	**1.252**	**1.066**	**1.024**	**1.000**	**0.8599**

Table S2. Calculation of the Competitive Index – bronchoalveolar lavage

**TIGR4*lux* vs TIGR4*lux*Δ*rr09***		**TIGR4*lux* vs TIGR4*lux*Δ*hk09***		**TIGR4*lux* vs TIGR4*lux*Δ*tcs09***	
**24 h**	**48 h**	**24 h**	**48 h**	**24 h**	**48 h**
0.000000	1.000000	0.696968	0.522248	2.708325	0.002445
1.312501	0.928570	2.599928	0.824072	0.858973	1.000000
0.491329	5.000000	3.421041	0.000000	0.750002	0.666600
1.000000	1.000000	2.735302	0.000000	0.096345	1.705882
1.384626	1.940300	0.067285	0.226415	3.499978	0.034479
0.124694	0.333330	1.238086	0.400000	0.708325	> 3
0.333300	0.052627	0.620553	0.025638	1.417584	0.857144
2.596159	0.750002	3.571429	0.116667	0.999963	0.052627
5.50000	0.181818	2.208331	0.000000	0.776311	0.230900
1.565891	0.538461	3.360013	0.354166	1.303027	0.052627
0.888413	> 3	1.190476	1.365854	0.416663	1.000000
2.760873	0.968744	1.249995	3.199979	0.999850	1.545451
3.321440	2.586225	0.200000	0.683330	2.043461	> 3
0.960782	1.924241	1.296294	> 3	2.083325	16.665000
**Median:**					
**1.156**	**0.8393**	**1.273**	**0.3771**	**0.9999**	**0.9286**

Table S3. Calculation of the Competitive Index – lungs

TIGR4*lux* vs TIGR4*lux*Δ*rr09*		TIGR4*lux* vs TIGR4*lux*Δ*hk09*		TIGR4*lux* vs TIGR4*lux*Δ*tcs09*	
**24 h**	**48 h**	**24 h**	**48 h**	**24 h**	**48 h**
2.500009	0.499998	3.000000	1.072916	1.285716	0.000495
0.004785	> 3	0.474967	0.662497	0.892563	0.064195
1.021281	1.000000	> 3	0.230769	0.485980	0.575758
1.000000	17.499025	0.643192	0.399999	0.023809	0.000266
0.442307	0.541095	0.077821	13.750318	0.165311	0.002132
36.00400	0.096000	0.330509	0.008215	0.166664	4.750000
1.314811	0.856287	1.875012	0.015269	0.555553	4.583331
1.764727	0.584268	1.000000	0.006589	0.032258	0.139286
0.328225	0.754386	2.376630	0.307692	1.475412	0.403588
0.617312	0.428572	1.500000	0.216931	0.000000	0.666667
0.169687	4.933320	0.063830	0.160950	4.998800	1.200001
2.607841	0.111111	0.274298	8.833300	0.111100	1.210521
0.573499	5.555533	1.156629	0.554349	0.470588	0.533330
> 3	3.073186	0.084416	2.894747	1.000000	0.142856
**Median:**					
**1.011**	**0.8053**	**0.8216**	**0.3538**	**0.4783**	**0.4685**

Table S4. Calculation of the Competitive Index – blood

**TIGR4*lux* vs TIGR4*lux*Δ*rr09***		**TIGR4*lux* vs TIGR4*lux*Δ*hk09***		**TIGR4*lux* vs TIGR4*lux*Δ*tcs09***	
**24 h**	**48 h**	**24 h**	**48 h**	**24 h**	**48 h**
0.026313	0.366012	1.000000	> 3	0.302325	0.000337
1.000000	0.224189	0.697337	0.625000	0.352941	0.024911
0.999999	0.000000	1.000000	1.000000	1.103438	0.307692
0.000000	2.070800	5.000000	1.000000	0.008621	0.000230
0.029411	0.367431	0.022280	1.068966	0.593176	0.001473
1.133320	> 3	0.467479	0.000000	0.002865	4.222200
1.489797	1.166665	0.000000	0.018348	0.857144	7.764797
0.000000	0.083798	1.000000	0.000000	1.000000	0.047847
0.851850	0.687152	0.321284	0.317391	1.533332	0.251967
0.024622	0.459459	1.475412	0.204663	0.025638	0.905108
0.138421	3.199979	0.000000	0.178451	1.000000	1.000000
0.272729	0.095994	0.354838	2.296289	1.000000	1.599993
0.671643	2.739837	1.298848	0.409091	0.529412	0.594061
> 3	> 3	0.012346	12.916650	1.000000	1.000000
**Median:**					
**0.4722**	**0.5733**	**0.5824**	**0.5170**	**0.7252**	**0.4509**

Table S5. Calculation of the Competitive Index – brain

**TIGR4*lux* vs TIGR4*lux*Δ*rr09***		**TIGR4*lux* vs TIGR4*lux*Δ*hk09***		**TIGR4*lux* vs TIGR4*lux*Δ*tcs09***	
**24 h**	**48 h**	**24 h**	**48 h**	**24 h**	**48 h**
1.000000	0.312500	11.333300	0.536424	1.312508	0.002278
2.000300	0.018518	0.754098	0.486488	6.000000	0.624997
> 3	1.000000	1.000000	1.000000	1.120000	0.333330
0.000000	0.490566	0.548377	0.199998	1.769188	0.250000
0.519998	0.225071	0.333333	1.033330	1.500000	0.012657
2.3333	2.400002	0.099990	> 3	1.000000	0.020406
1.785754	1.000000	0.000000	1.000000	0.863636	3.789494
3.190471	0.249998	0.238086	1.000000	0.000000	0.020406
4.000043	0.7743889	0.902434	0.087500	0.575755	0.480767
0.000000	1.000000	0.209801	0.152174	1.166667	> 3
0.200012	0.473684	0.656254	0.025532	2.499775	1.000000
0.888866	0.052631	0.750003	1.999985	0.655167	1.699995
7.666650	> 3	1.327578	0.358488	0.916650	0.290779
0.900000	0.398601	0.000000	1.062497	5.000000	1.000000
**Median:**					
**1.393**	**0.4821**	**0.6023**	**0.7682**	**1.143**	**0.4070**
